# An integrated understanding of the complex drivers of emergency presentations and admissions in cancer patients: Qualitative modelling of secondary-care health professionals’ experiences and views

**DOI:** 10.1371/journal.pone.0216430

**Published:** 2019-05-02

**Authors:** Hong Chen, Julie Walabyeki, Miriam Johnson, Elaine Boland, Julie Seymour, Una Macleod

**Affiliations:** 1 Academy of Primary Care, Institute of Clinical and Applied Heath Research, Hull York Medical School, University of Hull, Hull, United Kingdom; 2 Wolfson Palliative Care Research Centre, Institute of Clinical and Applied Heath Research, Hull York Medical School, University of Hull, Hull, United Kingdom; 3 Queen's Centre for Oncology and Haematology, Castle Hill Hospital, Hull and East Yorkshire Hospitals NHS Trust, Hull, United Kingdom; University of Mississippi Medical Center, UNITED STATES

## Abstract

The number of cancer-related emergency presentations and admissions has been steadily increasing in the UK. Drivers of this phenomenon are complex, multifactorial and interlinked. The main objective of this study was to understand the complexity of emergency hospital use in cancer patients. We conducted semi-structured interviews with 42 senior clinicians (20 doctors, 22 nurses) with diverse expertise and experience in caring for acutely ill cancer patients in the secondary care setting. Data analysis included thematic analysis and purposive text analysis to develop Causal Loop Diagrams. Our Causal Loop Diagrams represent an integrated understanding of the complex factors (13) influencing emergency hospital use in cancer patients. Eight factors formed five reinforcing feedback loops and therefore were high-leverage influences: Ability of patients and carers to self-care and cope; Effective and timely management of ambulatory care sensitive conditions by primary and community care; Sufficient and effective social care for patients and carers; Avoidable emergency hospital use; Bed capacity; Patients accessing timely appropriate specialist inpatient or ambulatory care; Prompt and effective management and prevention of acute episode; Timely and safe discharge with appropriate support. The loops show that reduction of avoidable hospital use helps relieve hospital bed pressure; improved bed capacity then has a decisive, positive influence on patient pathway and thus outcome and experience in the hospital; in turn, better in-hospital care and discharge help patients and carers self-care and cope better back home with better support from community-based health and social care services, which then reduces their future emergency hospital use. To optimise acute and emergency cancer care, it is also essential that patients, carers and other clinicians caring for cancer patients have prompt access to senior cancer specialists for advice, assessment, clinical decision and other support. The findings provide a useful framework and focus for service planners aiming to optimise care.

## Introduction

**Fig 1:**
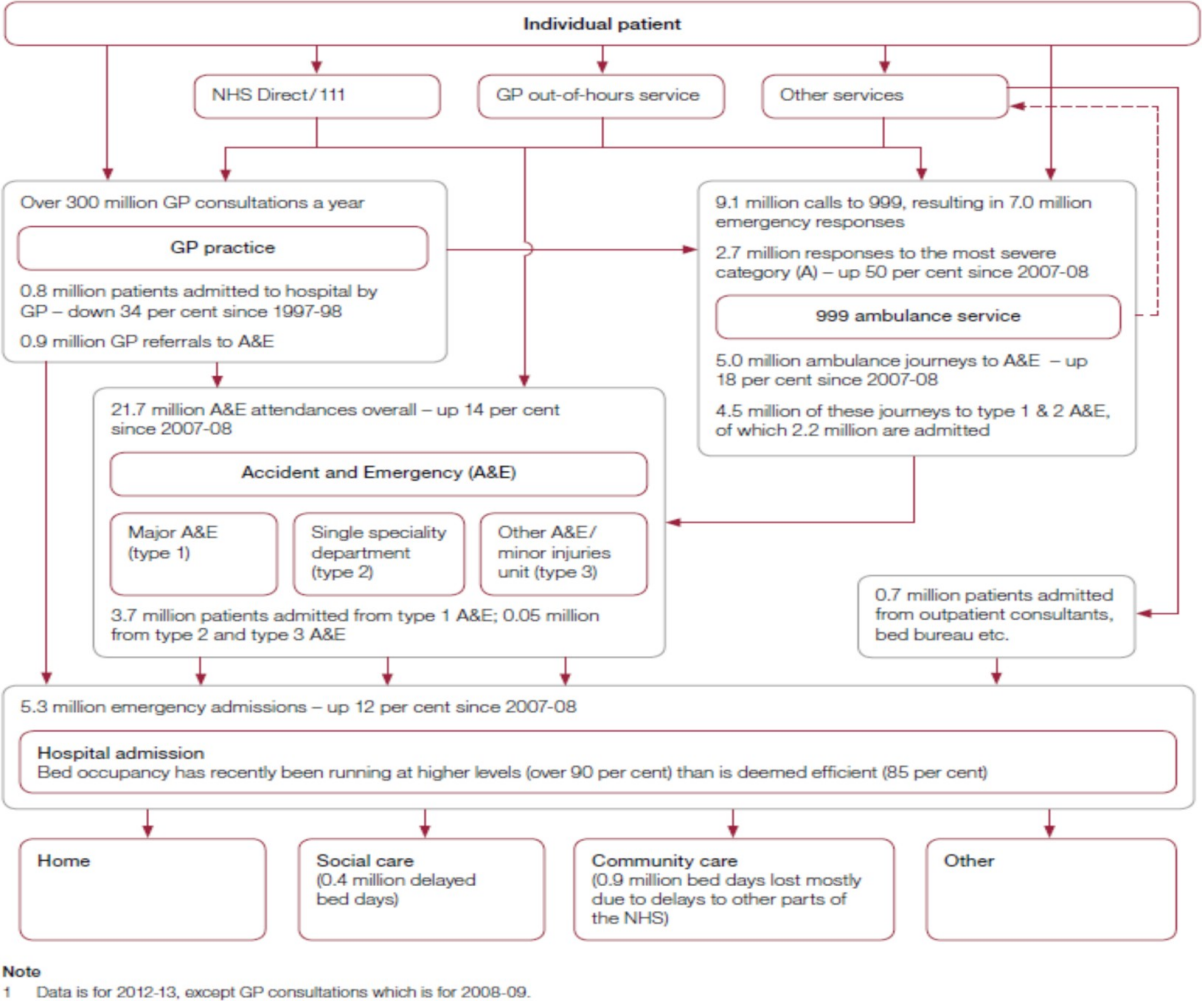
Patient routes that may lead to an emergency admission to hospital in England. National Audit Office (2013) Emergency admissions to hospital: managing the demand. London, UK: National Audit Office. https://www.nao.org.uk/report/emergency-admissions-hospitals-managing-demand/.

Avoiding unnecessary emergency admissions and managing those that are admitted more effectively is a major concern to the National Health Service (NHS) in England [1]. This is not only because of the costs associated with these admissions, but also because of the pressure and disruption they can cause to elective healthcare and, not least, to the individuals admitted. The number of emergency admissions in England has grown by 42% over the last twelve years (2006–2018) despite considerable effort to reduce it [2]. With a reduction in acute hospital beds, earlier English health policy focused on reducing emergency care demand by improving other parts of the healthcare system, i.e. primary and community care, social care, informal support and lay self-care [2,3]. Evidence suggests persisting scope for doing so [2]. However, even with the most effective services outside of hospital, hospitals will still be faced with increasing pressures. This is due to a sharp rise in the number of emergency admissions for patients with more severe or complex needs- whose care can be challenging outside hospital, such as those with cancer [4] and multiple health conditions [2]. Hospitals have attempted to manage the pressures by reducing waiting times in Accident and Emergency (A&E) department and lengths of stay in hospital, and improving outcomes for patients admitted to hospital [1,2]. Furthermore, the urgent and emergency care system in England is complex; and there are multiple pathways to emergency attendances and admissions: patients can be admitted to hospital via A&Es, walk-in centres, GP referrals directly on to the hospital ward and other routes [1], as demonstrated in Fig 1. The effective management of the flow of patients through the health system is also at the heart of reducing unnecessary emergency admissions and managing those patients who are admitted. The overall policy is therefore oriented towards involving all parts of the health system and joining up effort to enable people to remain in their own homes as long as possible while ensuring that admissions to hospital are appropriate and as short as possible [1,3]. In practice, primary, community and social care can reduce admissions through improving management of long-term conditions; ambulance services can reduce conveyance rates to A&E departments by conveying patients to a wider range of care destinations; hospitals can reduce emergency admissions by ensuring prompt initial senior clinical assessment, prompt access to diagnostics and specialist medical opinion; and once admitted, hospitals working with community and social care services can ensure that patients stay no longer than is necessary and are discharged promptly [1]. In summary, health policy and practice have started to acknowledge that causes for and solutions to increasing pressure on emergency hospital care are complex, multifactorial and interlinked. Yet, research evidence is still lacking regarding what complex factors are at play and how such factors interact and impact emergency hospital use, with research largely focused on discrete factors only.

The number of emergency admissions in cancer patients has also been increasing [5,6] with most patients presenting to and admitted *via* A&E [6,7]. Cancer patients often develop acute problems either due to cancer or its treatment, requiring an urgent response [4,6,8,9]. This is compounded by the increased incidence of cancer in older people and associated comorbidities. For acutely ill cancer patients, prompt and correct management in the most appropriate setting is critical [6]. However, such patient management is often complex, requiring interaction between a number of professionals and specialties. Therefore, timely and appropriate clinical decision-making and coordination of care may be difficult. The risk is that patients receive inappropriate care in the wrong setting, with consequential adverse clinical outcomes.

The rising burden of emergency cancer care has spurred development of specialised services for acutely ill cancer patients. Some urgent care centres (or A&Es) solely for cancer patients have emerged, mostly in the US [10–15]. Service configuration varies in team composition, referral process, adult or children, working hours, nurse practitioner- led or consultant-led. Initial evidence [10,12–15] suggests that the most significant benefit of these centres was the reduction of A&E visits for oncology-related symptom management. This is because A&E providers were ill-equipped to address common cancer-related symptoms such as pain, vomiting, or bowel issues in immune-compromised patients. Patients in these centres were often seen within minutes of arrival, diagnosed and managed more promptly resulting in faster symptom relief, compared to usual A&E care. Admission rates were much lower in these centres than in A&E with more patients effectively treated on an ambulatory basis, which reduced cost of care in both A&E and inpatient units. In England, similar services have also developed, e.g. specialist admission units in tertiary cancer centres and acute oncology services in acute general hospitals with an A&E [6,11]. However, there is little research evidence regarding the benefits of such services. A study conducted in the North West of England found that such services may improve communication across clinical teams, enable rapid specialist oncology review, reduce hospital stay, and increase understanding of oncology emergencies and their treatment [16]. In 2016, we conducted a study to explore cancer patients’ and carers’ views and experiences of emergency admissions and subsequent inpatient care in a hospital trust in the North East of England [4]. We found that locally, if cancer patients were still on active treatment and presented acutely or as emergencies with treatment side effects, they were most often directly admitted to an oncology ward following specialist advice, review and triage; and that subsequently they experienced outstanding specialist inpatient care. Although the findings suggested that the local tertiary cancer centre provided specialised emergency cancer care services which benefited patients and carers, it was not clear from patients’ and carers’ perspective how such services were organised and how they worked. We also identified gaps in health care in the community, but it was not clear whether this contributed to patients’ admissions. Moreover, the study raised new questions regarding pathways to emergency presentations and admissions for other types of cancer patients and subsequent hospital care they receive in other (non-cancer) parts of the local hospital system. These questions can be best answered by professionals who directly care for cancer patients on emergency care pathways.

Drawing on qualitative interviews with 42 (mostly senior) clinicians with diverse expertise and experience in caring for acutely ill cancer patients in the secondary care (hospital) setting, we aimed to a) map cancer patients’ pathways to emergency hospital care (presentations and admissions) locally; b) describe the specialised emergency cancer care services provided by the local tertiary cancer centre, and understand how they benefited cancer patients on various pathways- including direct presentations and admissions to the cancer centre and those to the local acute hospital *via* A&E; c) explore factors influencing emergency hospital use in cancer patients that are related to all parts of the cancer care system, and map the interactions and feedback loops among these factors to facilitate a whole-system, integrated understanding of the dynamics in emergency cancer care.

## Methods

This is a qualitative study of secondary-care health professionals’ experiences and perceptions of reducing and managing emergency presentations and admissions for cancer patients with a confirmed diagnosis. This is not a study about patients who are diagnosed with cancer within the context of an emergency admission. Ethics approval was granted by the ethics committee of Hull York Medical School on 8th February 2017 (Ref 17 01).

### Setting

**Fig 2:**
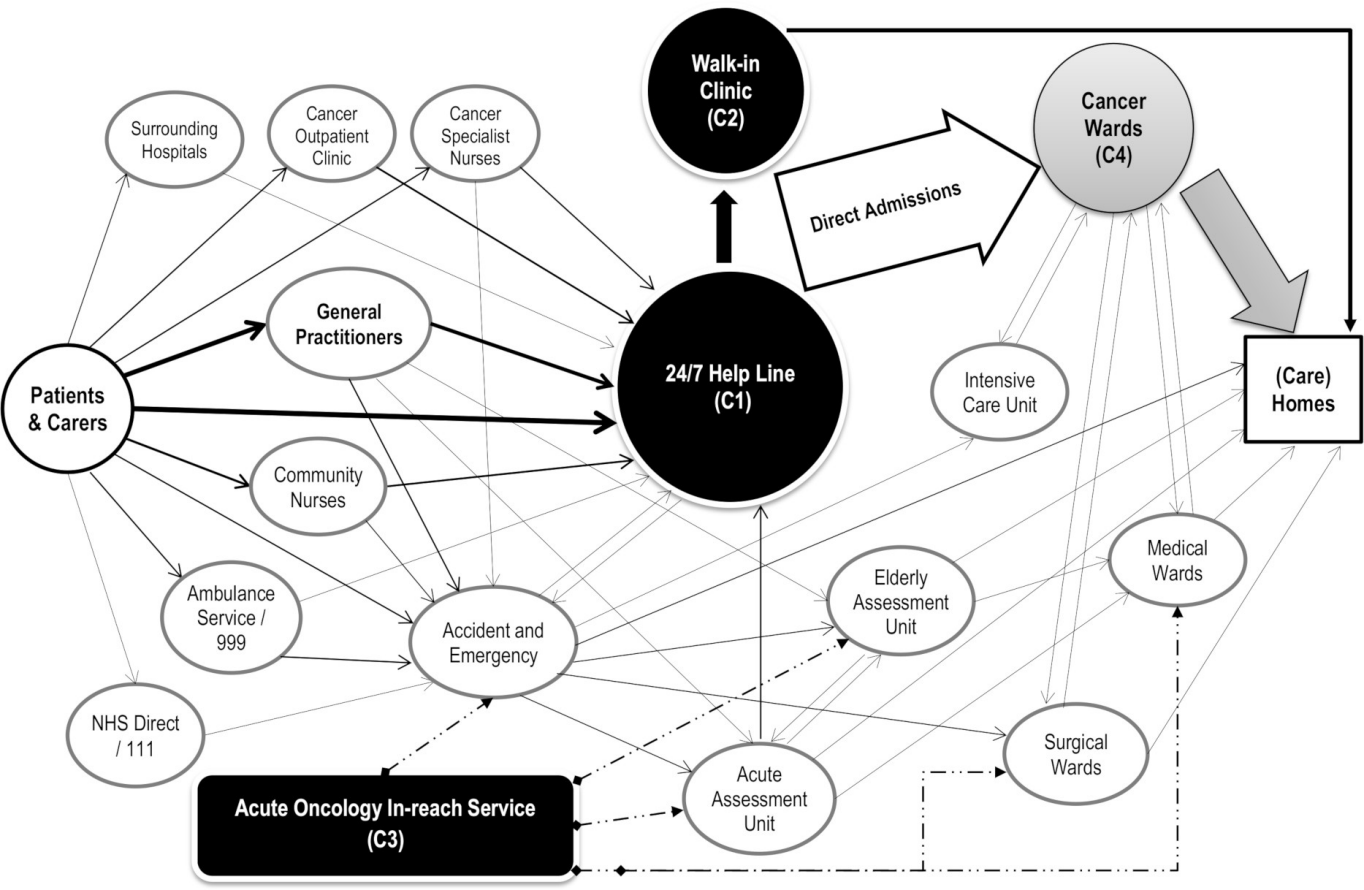
Emergency presentation/admission pathways in cancer patients. NHS Direct/111: NHS health advice and information service; Acute Assessment Unit: acute medical assessment/short stay unit (maximum 48h stay); Community Nurses: district nurses and specialist palliative care nurses; Cancer Outpatient Clinic: scheduled oncology or hematology outpatient appointments; Elderly Assessment Unit: acute frailty assessment/short stay unit (maximum 48h stay). This trust has two sites, with all oncology and hematology services and Intensive Care Unit at one site and A&E, Acute Assessment Unit and Elderly Assessment Unit at the other site; and there are medical and surgical wards at both sites.

The study was conducted in a hospital trust in the North East of England. The Trust operates from two main sites (two hospitals), with its tertiary cancer centre at one site and the major A&E at the other. The cancer centre serves a population of approximately 1.2 million people in a mixed urban/rural environment. Fig 2 shows the main health services that cancer patients in this trust could access in the primary, community and hospital settings, and possible routes through which they could be admitted to hospital as an emergency. Despite some convoluted pathways (often via A&E), most emergency attendances and admissions among cancer patients were directly managed by the cancer centre through its specialised emergency cancer care services (C1-C4).

### Participants

The research team is multidisciplinary, consisting of five academic researchers (HC, JW, MJ, JS, UM) and one clinical researcher (EB). HC has a background in public health and JS is a medical sociologist. JW, UM and MJ have a clinical background in primary care (JW, UM) and palliative care (MJ) respectively, but they are not employed in the participating trust. EB is the only one employed within the participating trust- as a palliative care consultant. Drawing on such diverse (outsider to insider) knowledge, we were able to identify key specialties, services and clinicians directly involved in the care of acutely ill cancer patients in the trust, and other players in the community (e.g. GPs, community nurses, patients and carers, social services). However, due to resource and time constraints, we made a decision to focus exclusively on secondary-care professionals in this study- to achieve an in-depth understanding of/around this part of the cancer care system. This is justifiable because in England secondary care is the core component of the cancer care system, i.e. once diagnosed patients are under the care of hospital specialists for the majority of their cancer trajectory, and the role of primary and community care is subsidiary. Nonetheless we were aware that the views of other stakeholders may differ from and thus supplement those of secondary-care professionals because of their unique positions in the cancer care system. We acknowledge this as a limitation (see implications of this in Limitations).

Participants were recruited purposively [17] to ensure that they were key clinicians directly involved in the care of acutely ill cancer patients in the participating trust; and that their experiences were as diverse as possible, i.e. they were from different specialties and services on different emergency care pathways. JW invited 71 clinicians (by email, by telephone, face-to-face). Most of these clinicians were senior: they were consultants (attending doctors), and band seven and eight nurses (e.g. matrons, ward sisters, advanced nurse practitioners and clinical nurse specialists). 42 clinicians participated. The sample included 20 medical staff and 22 nursing staff. Most (18 doctors, all nurses) were senior and some had both clinical and managerial responsibilities (e.g. clinical lead, clinical director). There were 17 men and 25 women from the following specialties: oncology and haematology (17), palliative care (3), elderly care (5), acute care (9), and others (8). 29 clinicians did not respond, including 12 nurses (most were senior) and 17 consultants from oncology and haematology, acute care, elderly care, palliative care and other specialities.

### Data collection

As discussed above in Introduction, our previous study [4] conducted in the same hospital trust raised questions to be answered from professionals’ perspectives. This combined with literature review, multidisciplinary team discussions and two pilot interviews informed the interview topic guide. The main topics included: a) existing processes of and pathways to emergency presentations and admissions for cancer patients; b) avoidable and unavoidable admissions with reasons; c) existing practices and suggestions to reduce avoidable and manage unavoidable ones in secondary care; and d) other factors influencing emergency hospital use in cancer patients, including the role of patients and families, community based care and wider issues.

42 semi-structured interviews were conducted by JW between March and September 2017. Data saturation was reached [18]: no new ideas emerged and recurrent themes became established. Participants gave written informed consent. All were interviewed face-to-face at their preferred place and time. Interviews, lasting between 11 and 65 minutes (average 23 minutes), were audio-recorded with consent.

### Data analysis

The interviews were transcribed verbatim and anonymised with a unique ID code and distinguishing features were removed. HC analysed the data with coding, theme and model development overseen by all members of the research team. Our multidisciplinary research team met regularly at every stage of the analysis and had extensive, reflexive and critical dialogues about how the ideas expressed by interviewees and identified in the transcript were related to pre-existing concepts and theories from each discipline, and to the real problems that the study was addressing. This helped to ensure that the main analyst has not drawn exclusively from the data that confirm her presumptions. The perspectives of colleagues from other disciplinary backgrounds also added analytic depth to data interpretation. This kind of team effort improved validity of interpretation and enhanced the credibility and relevance of the findings.

The analysis was composed of first, thematic analysis, and then purposive text analysis to develop Causal Loop Diagrams (CLDs). NVivo11 (qualitative data management software) was used to manage data and Vensim PLE (system dynamics software) used to produce CLDs.

In the thematic analysis, interview transcripts were analysed to categorise the recurrent or common themes [19]. The analysis was deductive: it was grounded in data, informed by concepts or issues emerging from the data, and *a priori* issues- those introduced into the interviews as informed by the research questions. This provided the basis for mapping cancer patients’ pathways to emergency hospital care and identifying discrete influencing factors.

Further, our data contained very rich depictions of the complexity of the matter under study. In this case, complexity stemmed from a combination of the complexity of the disease itself and that of the emergency cancer care system. The data resonated with the perception of health systems from a Systems thinking perspective. That is, health systems are complex adaptive systems “because they involve multiple interacting agents, the context in which they operate keeps changing, because the manner in which things change do not conform to linear or simple patterns, or because elements within the system are able to learn new things, sometimes creating new patterns as they interact over time” (p2) [20]. Although thematic analysis would allow for rich, detailed and complex description of such data, we needed an analytical tool that would help us understand and visually display intricate processes and root causes of a complex problem, and a complex system with its parts, relationships among the parts and the behaviour of the entire system.

Systems thinking (science) has been applied increasingly to health and health systems research because its core aim is to understand and communicate linkages, interactions, feedbacks, and processes between the elements within some notion of a whole entity [20]. Theories, methods and tools in Systems thinking are each designed to address complex problems, such as Systems Dynamics. It uses a set of tools to capture and understand the behaviour of complex systems over time [21]. CLDs is a common Systems Dynamics tool that produces qualitative illustrations of mental models, focused on highlighting causality and feedback loops [22] and has been used in health service research [23–27].

Following thematic analysis, HC used Kim and Andersen’s [28] purposive text analysis to elicit CLDs from the data. This method employs an entirely inductive approach to identify problems, key variables, and their structural relationships from raw qualitative data. This was viable for a number of reasons. First, participants were key decision makers or stakeholders in the system under study, and provided sophisticated, expert knowledge of the system. Second, the data captured the participants’ focused discussions on the system and the problem at hand, including rich causal and dynamical depictions. Third, it could be reasonably assumed that the mental models of the participants were revealed because their discussions appeared to be frank and unfeigned. This approach is important when the text data are neither collected by the modeller nor intended to be used for the system dynamics modelling purpose, as in our case. It provides specific, analytical steps and documentation methods, to ensure CLDs are grounded in text data and their linkages to original data segments are traceable. Thus, it helps the modeller to build confidence in the soundness and usefulness of the models generated from the qualitative data. The core analytical steps included: a) identifying data segments that consisted of one argument and its supporting rationales; b) from each data segment, identifying the cause variable, effect variable, and the polarity of the relationship; c) using simple words-and-arrow diagram to represent each causal relationship; d) collecting and merging the words-and-arrow diagrams into a collective CLD- collapsing similar variables using a common variable name, with the aid of Vensim PLE. Variables identified through the purposive text analysis mostly overlapped with the discrete factors influencing emergency hospital use—derived from the thematic analysis. They were combined to refine the CLDs and the supporting narrative about the CLD variables.

## Findings

### Specialised urgent and emergency cancer care

*In whole we try to manage all of our own erm workload so we try very hard to avoid people going into medical pathways*, *so lots of patients will come into the [cancer centre] who in other comparable units would not necessarily be admitted under erm under haematology or oncology they’d end up in medical pathways*. (020)

The participants described the cancer centre’s specialised emergency cancer care services and as a result, cancer patients’ pathways to urgent and emergency care locally, as shown in Fig 2. The services mainly consisted of a 24/7, rapid-access helpline for advice, triage and admission (C1), a walk-in clinic (C2), an acute oncology in-reach service (C3), and four cancer wards (C4). The helpline (C1) was run mainly by senior nurse practitioners, with support available from mid-grade (registrar) and senior (consultant) doctors. They a) gave patients, carers and community-based professionals advice on the (self) management of symptoms and service utilisation; b) carried out telephone assessment and triaged patients to the walk-in clinic or alternative services; c) managed bed space and arranged emergency admissions to the cancer wards. The walk-in clinic (C2) had a capacity of reviewing 20–30 patients a day by on-call registrars during normal working hours and fewer patients by senior nurses during out-of-hours periods; these patients were either discharged the same day or admitted if necessary. The four cancer wards (C4) had 99 consultant-led beds. Locally, the majority of emergency attendances and admissions among cancer patients with a known diagnosis were directly managed by the cancer centre, following self-referral or referral by a professional, as demonstrated in Fig 2. A small number of patients appropriately or inappropriately attended or were admitted *via* A&E to other (non-cancer) short-stay units and wards, i.e. “medical pathways”. For these patients, an acute oncology team (consultants and nurse practitioners) provided an in-reach service (C3) during the week to support other specialities to care for them. In addition, a specialist palliative care consultation team based in the cancer centre contributed to the management of emergency attendances and admissions.

### Complexity and dynamics of the drivers of emergency cancer care

**Fig 3:**
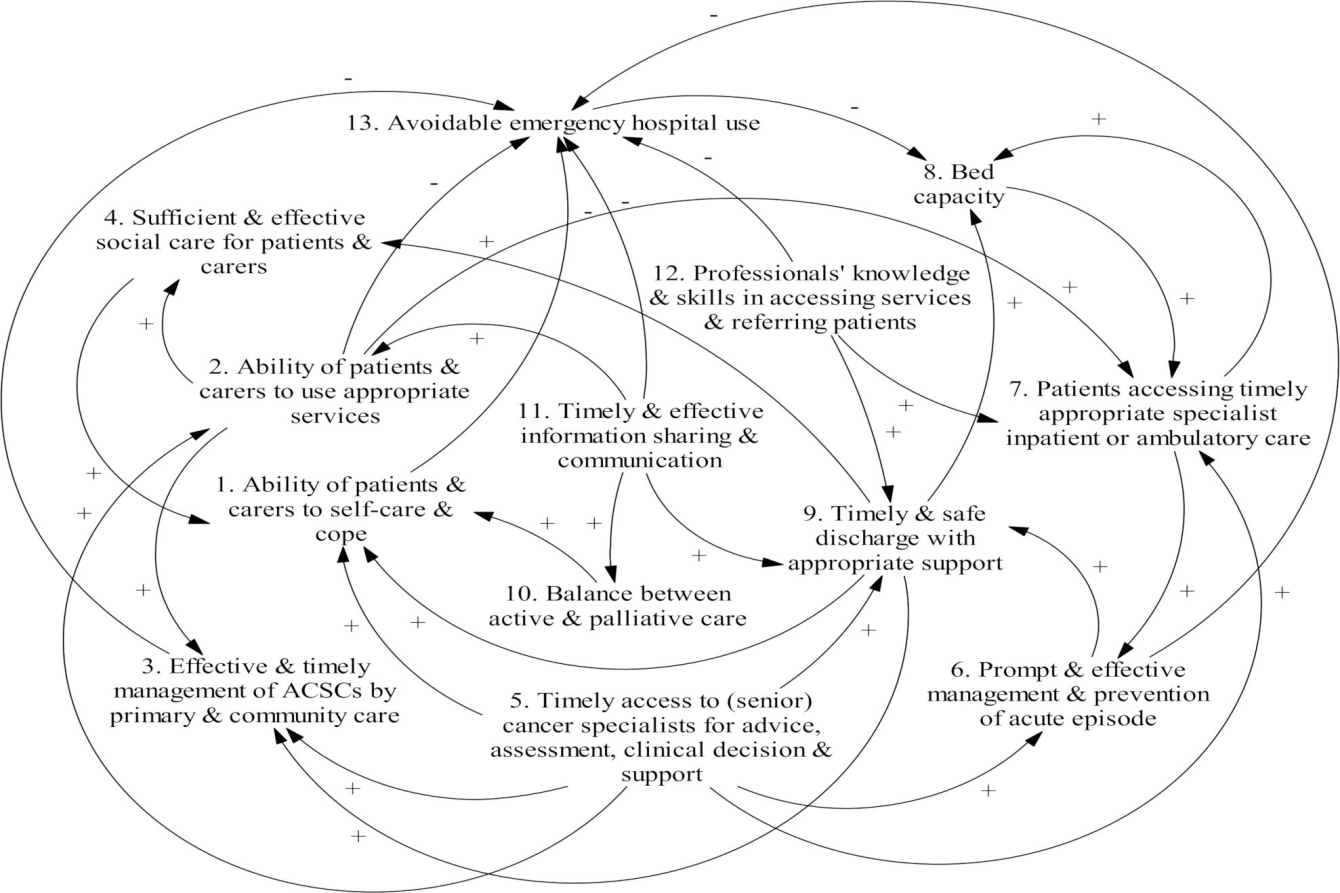
CLD illustrating complexity in the reduction and management of emergency attendances and admissions in cancer patients. CLDs are composed of two components: variables and influences (links). An influence has direction shown by an arrow and an indicator as to whether the influenced element is changed in the same (+) or opposite (−) direction as the influencing element.

The participants also identified factors that played a role in a) keeping patients out of hospital as appropriate and/or b) improving clinical outcomes and patient experience for those attending or being admitted to hospital. These related to patients and carers, community-based health and social care services, hospital based services, and interfaces between different parts of the care system. Using the CLDs method, we mapped the major interconnections and interactions among these factors. This resulted in an integrated representation, a CLD (Fig 3), of the complex drivers of emergency cancer care as understood by the participants. It not only summarises the discrete influencing factors (CLD variables 1 to 13) but also demonstrates the interactions and most importantly, the feedback loops among these factors. We describe and explain each CLD variable first and finally the feedback loops.

#### 1. Ability of patients and carers to self-care and cope

*We’re dealing with a primarily elderly population*, *you know, self-caring and stuff like that can often be reasons for admission (001)*

Cancer treatments were delivered increasingly in the outpatient setting. Subsequently, patients and their carers needed to self-manage symptoms from the cancer and its treatment (and comorbidities) between outpatient appointments, while restoring or maintaining their general health and wellbeing. Their abilities to self-care varied. The less able were more likely to need urgent or emergency care, such as older people with co-morbidities and little support from family carers or social services, where potentially avoidable deterioration or disease exacerbations led to a need for urgent care. Psychological burden, relating to the demands and uncertainties of cancer, was often heavy on both patients and families. As lay people, they had to self-manage symptoms that were difficult even for professionals. Understandably, they felt anxious, helpless or frightened when faced with unexpected, unfamiliar or uncontrollable symptoms, particularly during the terminal phase. Panic therefore often triggered emergency hospital use (attendances and admissions), especially in patients living alone as well as carers witnessing patients in distress. Carer strain and burnout was also cited to have triggered emergency hospital use- as respite care for carers and a safer choice for patients.

#### 2. Ability of patients and carers to use appropriate services

*They don’t necessarily understand what all the different people do*, *so I can imagine as a carer*, *or a patient it’s then very confusing when you’ve got a problem who do you*, *who do you contact*. *(008)*

Patients and carers ideally should use the most appropriate services fit for their specific needs at the right time. This is crucial for both patient outcome and experience, and health service efficiency and cost saving. To achieve this, they needed to navigate the care system: the more complex their needs, the more complex and fragmented their care system was. Fig 1 and Fig 2 demonstrate the complexity of the current system. Without knowledge about services, or the skills to access them, patients defaulted to the most easily accessible and known service. For example, patients and carers who did not know or understand how and when to use community palliative care services or the cancer centre’s emergency care services turned to A&E instead.

#### 3. Effective and timely management of Ambulatory Care Sensitive Conditions (ACSCs) by primary and community care

*They need to improve the resources at the GP [family practitioner] level and the community so that the people are looked after in the community by their local doctors*, *are not coming for simple issues to hospitals*, *people come too frequently to A&Es and to oncology for issues which may not be relevant to hospital medicine at all*. *(033)*

There was a consensus that GP practices, district nursing and specialist palliative care teams were generally “stretched”, being “hugely under-resourced, under-staffed”. Hence, availability and accessibility of both long-term and urgent and emergency care were limited. For example, patients could get neither normal GP appointments nor urgent ones either in the surgery or as a home visit. Patients were not seen by district nurses or specialist palliative care nurses as frequently in long-term care or as promptly in crisis as they needed to be; urgent care was the least available and accessible during out-of-hours periods. Moreover, some GPs and district nurses lacked knowledge, skills and confidence in managing acute cancer-related conditions. These resource, capacity and skills issues led to emergency hospital use even among dying patients. To keep patients out of hospital, it was hoped that primary and community health care teams effectively manage ACSCs in a timely manner. Examples include: managing patients’ long-term conditions to prevent flare-ups; monitoring patients to identify and treat acute problems early to prevent crisis; responding to patients’ urgent needs more rapidly; referring patients to alternative community based services; and providing general holistic support for both patients and carers.

#### 4. Sufficient and effective social care for patients and carers

*There's certain areas where you wait longer for care packages because there's no carers available in those areas*, *so I think*, *I think definitely what happens in the community does have an impact on the hospitals because*, *because if there's not that service in the community where else are the patients going to go*? *(013)*

Similar resource, capacity and skills issues existed in social care services. There were a limited number of care homes and a shortage of professional carers in care homes or as domestic workers. This led to generally low-level, insufficient long-term care for patients and carers and delayed responses to their urgent needs. Besides, some professional carers were poorly trained with limited skills. In care homes, there was a shortage of nursing staff to support carers; and some of them also lacked knowledge, skills and confidence to deal with acute problems in cancer patients. Meanwhile, care home staff struggled to get timely support from primary and community health care teams. In an emergency, they tended to send patients to hospitals. Other social services were also sparse, for example, physio and occupational therapy, respite care for carers, home adaptations and equipment. Lack of the services to help with tasks of daily living meant that patients ended up with general health and functional declines or disease exacerbation, leading to avoidable hospital use “for social reasons”.

#### 5. Timely access to (senior) cancer specialists for advice, assessment, clinical decision and other support

*We do have the advice line and people ring up so*, *you know*, *everybody that rings up we don’t always admit*, *so there is a lot of triaging*, *there’s a lot of advice over the telephone*, *erm*, *there is a lot of times that we can say “you need to go to your GP because they can just give you this and then you’ll be absolutely fine” or we do have a… a system where patients can walk in*, *and we call it “the walk in clinic” so in outpatients… and they can get (*.*) twenty*, *thirty patients a day just walking in*, *getting advice*, *maybe getting some antiemetics and going home or whatever*, *erm*, *so we do have er*, *you know*, *a system where we do prevent a lot of admissions*, *we have that system in place*, *so*, *you know*, *the majority of people that are ringing up and we are admitting*, *erm*, *it is because they really do need to be in hospital*. *(038)*

In urgent or emergency situations, patients and carers had direct, rapid access to the cancer centre’s emergency care services- led by cancer nurse specialists and consultant oncologists. It functioned to prevent unnecessary hospital use by supporting patients, carers and community-based professionals to (self) manage acute symptoms in the community (the help line). It helped to improve clinical outcome and patient experience in the hospital by providing prompt and effective care: directly (the walk-in clinic and wards) or by supporting other specialities (the in-reach service). Despite its recognised success, it had limitations. The most prominent one was that the walk-in clinic, with inadequate staffing, skill mix (no general and acute medicine expertise), space and other resources, was unable to provide a 24/7 service for all cancer patients. Subsequently, the help-line often admitted patients following assessment over the phone without face-to-face rapid assessment. This sometimes led to unnecessary admissions. Some patients could and should have been treated on an ambulatory basis had the clinic had full capacity. It also meant that some patients had to go into “medical pathways” *via* A&E if input from general or acute medicine experts was needed.

Many stressed the importance of patient and carer education given by specialists: the better the education, the better their abilities to self-care and cope, and thus the less the admissions. For example, for patients on chemotherapy from which serious side effects and complications were to be expected, there were dedicated, specialist nurse-led educational sessions about warning signs and appropriate courses for action including using the right urgent and emergency care service.

It was considered crucial that patients had both continuing and rapid-access contact with a specialist nurse throughout their cancer course for ongoing advice, education and other support. Ongoing contact allowed early identification and management of problems to prevent crisis. This also applies to having access to senior doctors (see 9).

Timely access to senior cancer specialists for advice was also important for other clinicians; access options included *via* the cancer centre’s helpline or “Ask Haematology”- consultant-led email and telephone advice. Also, senior specialists helped community professionals optimize cancer care by providing training in active and palliative/end-of-life care. In the hospital, improved clinical outcomes and reduced admissions resulted from them proving timely support for junior clinicians and other speciality teams in clinical assessment and decision-making.

#### 6. Prompt and effective management and prevention of acute episode

*It’s better for the patients if they’re admitted directly to the [Cancer Centre] if they can be*, *and I think*, *you know*, *they are seen quite quickly and hopefully the*, *whatever the*, *the situation is would be dealt with very quickly*, *particularly if it’s an emergency like a*, *a neutropenic sepsis or a cord compression*, *you know*, *that the…*, *there isn’t a delay really in*, *in getting*, *um*, *the treatment and the scans that they would need*. *(002)*

Urgent and emergency hospital use was sometimes appropriate and inevitable, resulting from cancer and its treatment (e.g. spinal cord compression, neutropenic sepsis, effusions, gastro-intestinal bleeding). In such cases, the goal was to promptly and effectively control symptoms, stabilise acute conditions, and prevent further acute episodes. Most patients on the cancer centre’s specialised emergency cancer care pathways benefited from simpler (shorter) pathways with faster access to the most clinically appropriate specialist (ambulatory and inpatient) care, compared to those using A&E. Patients had the opportunity to be rapidly assessed and treated in the walk-in clinic. If they were admitted to the cancer wards, most were reviewed fairly quickly by on-call consultants who usually attempted to resolve the acute episode promptly without disrupting patients’ long-term treatment. If there was need to change any of the long term management of the patient, input from patients’ own consultant would be sought. The on-site, specialist palliative care team supported the cancer teams to manage complex pain and other symptoms in advanced cancer. Preventive measures were also taken in view of recurrence of the same symptoms and conditions or new occurrence of predictable ones. For the small number of cancer patients on “medical pathways”- appropriately or inappropriately, the acute oncology in-reach team helped to improve the timeliness and effectiveness in managing and preventing their acute episodes (see 7).

#### 7. Patients accessing timely appropriate specialist inpatient or ambulatory care

*Sometimes when you're admitted acutely*, *if you end up under the wrong specialty getting someone back to the right specialty is really difficult*, *you know*, *and you could almost do it faster by discharging them and saying go back to A&E and tell them these symptoms and then you'll end up under the right team*, *which*, *you shouldn't record that really but there we go*. *(021)*

Response time and access to appropriate expertise were regarded as critical in urgent and emergency care, especially to achieve “Prompt and effective management and prevention of acute episode” (see 6). Ideally, patients would attend an appropriate urgent and emergency care service (the cancer centre versus A&E), and then be discharged the same day or go on to an appropriate specialty bed for onward care. In reality, this was challenging. Some patients should have attended the cancer centre instead of A&E or *vice versa*, i.e. should have been assessed at the right place at the beginning. Avoidable referrals (transfers) thus ensued. Following acute assessment, sometimes there was delay or failure in referring (transferring) patients to the best place for onward care, for example, from “medical pathways” to the cancer wards or *vice versa*. Patients with complex symptoms due to comorbidities tended to have more complex pathways and lengthier journey in the hospital because of the complexity in both their medical condition and the hospital system (see Fig 2). They were at a higher risk of a) ending up in a less appropriate ward because it was more challenging to identify which pre-existing condition was causing the symptoms, or b) experiencing more delays with multiple transfers to different wards.

Various reasons were given regarding these problems, with bed pressure highlighted as the biggest problem (see 8). Another reason was that patients unknown to (not under the care of) an oncologist were not eligible for the cancer centre’s emergency cancer care services. It was also related to service utilisation by patients, carers or professionals, e.g. their knowledge, navigation skills and preferences (see 2 and 12). Besides, patient care involved various professionals and teams. Staff shortage and other capacity issues throughout the hospitals meant waiting lists (e.g. for tests, results, pharmacy, surgeons, radiologists, admin) and thus delays everywhere, which also contributed to delays in moving patients to the right places.

Under these circumstances, patients tended to have prolonged hospital stays and unmet needs, and thus worse outcome and care experiences. The prolonged journey with “lots of different moves, lots of different faces” (018) put patients at a higher risk of infections and functional loss (e.g. older patients and those on chemotherapy). Patients were cared for by clinicians without the relevant expertise and resources. This affected clinical decision making, risking inappropriate and possibly futile interventions and unmet needs. The in-reach service together with the palliative consult team helped to resolve these issues to some extent.

#### 8. Bed capacity

*If there aren't beds in the cancer ward they might get them to go through acute assessment and then they might be reviewing them as well but*, *you know*, *they might end up under the care of whoever in the*, *in this block has a*, *a bed and that's*, *you know*, *sometimes bed problems and bed pressures mean that we look after patients that would be better off looked after elsewhere*. *(016)*

Bed pressure prevailed everywhere, hindering patient movement- to the right care at the right time. It was to do with limited bed space, high bed occupancy, sub-optimal bed use, and limited staffing and other resources. Consequently, no bed available at the right place was the most often cited reason for patients attending and being admitted to a sub-optimal place. For example, when there was no bed in the cancer centre, patients sometimes were directed to A&E or they were transferred from A&E to AAU to wait for a cancer bed; patients without cancer care needs were admitted or transferred to cancer wards to wait for a medical or surgical bed they needed (see Fig 2). As such “the patient's got an extra step in their journey” (031) or more extra steps; they were not getting needed specialist treatment; and they were occupying the bed of another specialty- adding to *their* bed pressure. Also, as patients were waiting in a wrong bed, some became too unwell or unstable to be transferred to the right bed, particularly across two hospital sites. The common practice of “we look after patients that would be better off looked after elsewhere” (016) had consequences for both patient outcome and experience and onward bed capacity.

#### 9. Timely and safe discharge with appropriate support

*It’s about making sure everything’s on… is in place when the patients are discharged that you…they’ve gone through*, *er*, *you know*, *it’s a safe discharge*, *that they’ve spoken to everybody that they need to*, *the District Nurses*, *GP’s got the information*, *that they’ve gone through the… the tablets to take home*, *that… that the patient knows what tablets they’re taking*, *when to take them*, *you know*, *those are all (*.*) although it sounds (*.*) simple*, *and they all should happen (038)*

Safe and timely discharge with well-coordinated support in the normal place of residence was regarded as critical in preventing re-admissions. It means that a number of things needed to be in place before patients were “ready to go and safe to go” (038). First, patients needed to be “medically fit for discharge” as the result of prompt and effective management and prevention of acute episodes (see 6). Second, follow-up outpatient appointments had been arranged, prescription ready, and patients and carers educated about recovery, medication and other procedures (e.g. stoma care). It was important that consultants or registrars review patients regularly (scheduled appointments) in an outpatient clinic or that patients have open-access to them (flexible appointments) post discharge. Third, patients and carers’ support needs had been assessed and medical, nursing and social support services were effectively and sufficiently set up in the community. Failing any of these, patients might “bounce back in”.

Although faced with difficulties in setting up both health and social services, many reported social services were the most problematic because “a terrible amount of obstacles and horse trading goes on” (021). It was down to the resource and capacity issues- the same that caused the sort of avoidable admissions “for social reasons” at first (see 4). The social care crisis caused prolonged length of stay- the worst for those needing a care home placement, and also extra transfers and avoidable stays to wait for discharge (e.g. from an assessment or a short-stay unit to a ward), all adding to bed pressure. This could compromise patient outcome and experience (e.g. loss of mobility). It was particularly harmful to dying patients because “for a lot of them the window of being fit can be quite small..... waiting for discharge means that a proportion of them will never hit discharge” (021). Also, post discharge, sometimes patients and carers struggled to cope with what could be arranged for them and ended up back into hospital quickly.

Coordination with and handovers to multiple community teams entailed knowledge and skills in accessing services (see 12), and enormous amount of work because “there is a lot of, erm, you know, negotiating and telephone calls and ringing people and trying to get services set up” (038). Low staff levels meant that discharge process was not prioritised and optimised, causing delays and other problems. This was improved by having dedicated support personnel: “somebody who’s concentrating on getting that…those patients ready for discharge” (019). The dedicated staff (discharge liaison or coordinator) often had good knowledge of community services and communication skills, and “good links with a lot of our community colleagues” (038). They were able to start discharge planning and process much earlier and make more effort to optimize community support. They increased greatly the chance of success in discharging patients.

#### 10. Balance between active and palliative/End-of-life (EOL) care

*When patients have got to their sixth*, *seventh or eighth line of therapy and have been refractory to several of them you're pushing that patient too far and what they need is a*, *is a sensible clinician to sit them down and say you're at the end of your*, *of the line*, *we should not give you any more treatment because you're better off making the most of the time you have and not going through a treatment that may have some response in a third of patients but will have significant side effects and time in hospital in nearly a hundred percent of patients*. *(023)*

A large proportion of cancer-related emergency admissions resulted from complications or side effects of curative therapies. Some pointed out the role over-treatment played in emergency admissions. There was a tendency that patients were offered more treatment, more “hard-hitting” treatment and for longer. Far more focus was on treating even when patients had advanced cancer. This led to patients becoming frailer and sicker as cancer advanced; in turn, they were more likely to have complications and side effects and thus more emergency admissions “further down their cancer pathway”. As such, a need was identified to balance between treating and palliating: as cancer advanced, the focus should shift more and more towards palliating; at some point, “the treating” had to stop altogether so that patients could “make the most of the time [they] have”, instead of “dying in hospital because [they]'ve got neutropenic sepsis for treatment that was never going to work in the first place” (023). This required a culture shift from denial of and fight against death at all costs to acceptance of and preparation for it; and coming along with it a mindset that

*it’s just as important to give people erm a good death and find out what their concerns and wants and worries are and plan for the end of their life erm well in advance*, *as it is to always offer new treatments that may or may not add benefit*. *(020)*

#### 11. Timely and effective information sharing and communication

*I think there’s quite a lot of work still to be done in encouraging earlier recognition of when people are approaching end of life*, *earlier advance care planning*, *um*, *improving patients’*, *um*, *sorry*, *professionals’ communication skills and their confidence in having what can be quite difficult conversations*, *and that is then all backed-up by having the correct systems and processes in place for everyone to have the information*. *(008)*

It was highlighted that patient information did not flow reliably and promptly alongside patient movement throughout the care system. This compromised continuity, effectiveness and efficiency of care, and thus increasing the risk of avoidable hospital use. For example, ineffective documentation or sharing of discharge summary led to community teams failing to meet patients’ needs post discharge, leading to re-admission. There were various problems with the content, quality and quantity of information recorded on paper and electronic documents. For example, some templates were not designed to capture sufficient, relevant information; sometimes professionals failed to fill in the templates correctly or not at all. There were also barriers to communication which affected imparting or exchanging of information. For example, posted documents were delayed or lost; incomplete or inaccurate information was passed on through a third person (secretary or receptionist). There was an increasing reliance on IT to facilitate patient information flow and communication (to reduce errors and delays) and thus care integration. However, different electronic systems were used by different teams and providers in the same and different settings. These systems “don't talk to each other”- not connected and integrated. Often, with access to only their own systems, professionals could not share and access patient information across teams, providers and settings (e.g. between GP practices and hospitals). As such, the electronic systems had become a prominent problem in itself posing more barriers and creating more work in information recording and sharing.

Some “difficult conversations” between professionals and patients (and carers) were regarded as prerequisites for the prevention of some avoidable admissions and inappropriate treatment in the hospital. The first was “the honest conversations that they [specialists] should have about what people’s prognosis and outcomes really are and what the real potential benefits of treatment are” (020). Such conversations could help patients and carers develop realistic expectations regarding cancer and its care, and their future, which then could open the door to planning for more balanced care (between treating and palliating) and making end-of-life plans such as advanced care planning (ACP) and do not attempt cardio-pulmonary resuscitation status (DNA-CPR) (see 10). Second, a consensus was reached that cancer patients with advanced disease approaching end of life should have been “kept comfortable at home “if they so wished; and that when they were admitted, they should not have been given “treatment that might prolong their life for a couple of days” while “opening up the window for more suffering, not allowing them to die”(040). These situations were believed to be avoidable if ACP and DNA-CPR were discussed, documented and shared effectively in a timely manner.

#### 12. Professionals' knowledge and skills in accessing services and referring patients

*Sometimes we don’t always know what’s available and it’s how*? *in all my experience that I’ve got*, *I’m trying to find out cus*, *I’m needing to access different things in a different remit and sometimes how you access it in an outpatient setting is different to the wards*. *(025)*

It was acknowledged that professionals themselves did not necessarily know about other services that were available: what these services offered, how they worked, their limitations and strengths, and how to access them or work with them. This included not knowing about services in other settings (typically) but also those in the same setting offered by different specialties or teams. For example, it was not clear to other specialties what kind of patients could be referred to the cancer centre; or cancer specialists did not know about or how to access other specialist ambulatory care services to which cancer patients could be referred. Many admitted that they had little knowledge about or limited skills in accessing the services available in the community while pointing out that GPs might not know all the hospital services and the most appropriate one they should refer patients to in a particular situation. These happened because health and social care services were not only complex but also adapting and constantly changing. Thus there was a need for professionals to improve their knowledge and skills in accessing services as well as design clear pathways for signposting and referring patients to alternative services.

#### 13. Avoidable emergency hospital use

*If patients were better educated or had a better link to a GP*, *or a specialist nurse*, *or a consultant*, *or outpatients*, *they could be the avoidable ones*. *(007)*

Emergency attendances and admissions that were regarded avoidable resulted directly or indirectly from the factors described above (1–12). Reducing avoidable hospital use was therefore shared responsivity of all players and parts of the cancer care system.

#### Feedback loops

**Fig 4:**
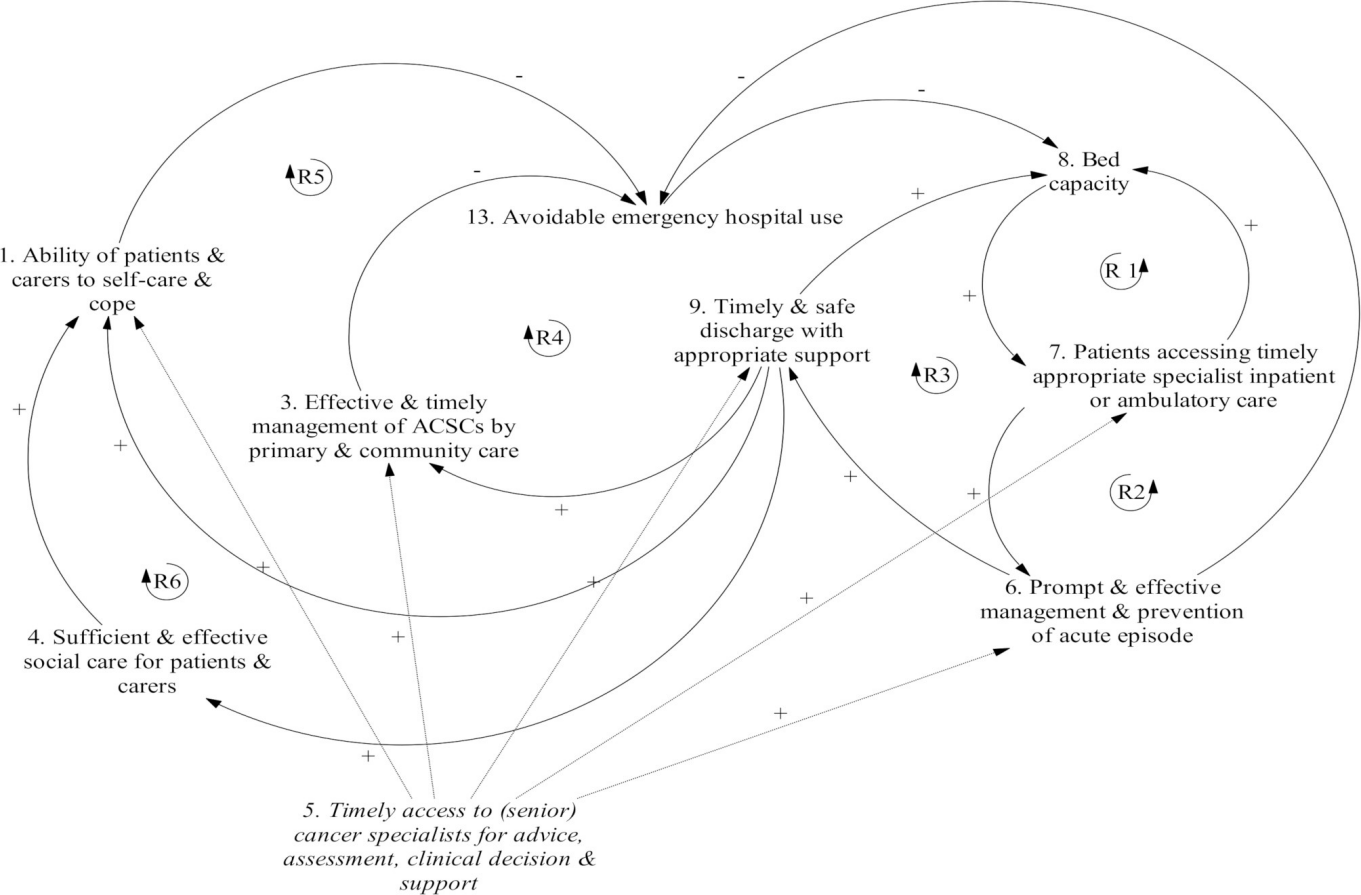
Reinforcing feedback loops in reducing and managing emergency attendances and admissions. Feedback loops occur when arrows connect a variable to itself through a series of other variables. Reinforcing loops, which indicate that variables have an overall amplifying effect, are labelled with an “R” and a loop symbol. Balancing loops, which indicate that variables have an overall dampening effect, are labelled with a “B” and a loop symbol. The loop symbol is either clockwise or counter-clockwise, depending on the direction in which the loop is read.

As a whole, Fig 3 shows how an event or action to change a driver (variable) will have a ripple effect and bring about unintended or unpredicted consequences- through interactions and feedback loops among variables. Furthermore, five reinforcing feedback loops are revealed- lifted from Fig 3 and illustrated in Fig 4 for ease of understanding. Reinforcing loops amplify change and may represent *virtuous cycles* where positive (favourable) change is amplified and negative (undesirable) factors decay or *vicious cycles* where negative changes are amplified and positive changes decay. Here we focus on potential virtuous cycles.

Reinforcing Loop 1 (R1) shows that it frees beds for patients in real need to get patients who have attended the cancer centre or A&E to the appropriate specialist inpatient or ambulatory care at the right time- without delays, unnecessary referrals and stays. In turn, increased bed availability speeds up the process of getting subsequent patients to the appropriate bed quickly. With improvement in getting patients in a timely manner to the right specialist team, patients’ acute problems can be managed more promptly and effectively with preventive measures in place. This then prevents future emergency hospital use (R2) and speeds up discharge thereby reducing length of stay (R3)–both of which in turn frees beds for subsequent patients and enabling them to have appropriate and timely care. Adding to R3, the more successful discharge is (speed, safety and coordination of care): a) the better patients’ medical and nursing care needs are met in the community (R4), and b) the better patients and carers are physically and mentally conditioned (e.g. no loss of mobility) (R5) and socially enabled (R6) to self-care and cope at home. As patients’ health care needs met and ability to self-care and cope improve at home, future re-attendances and re-admissions can be reduced, which then helps relieve bed pressure. The self-propagating ability of the loops can be strengthened or weakened by changing the factors that have direct or indirect effect on any factor on the loops (see Fig 3). In this regard the most significant factor is Timely access to (senior) cancer specialists for advice, assessment, clinical decision & support: it strengthens five factors on the loops- more than any other factors do, thereby augmenting these virtuous circles (see Fig 4).

## Discussion

We identified a model of comprehensive, specialised, urgent and emergency cancer care and unpacked how it optimised management of acutely ill cancer patients on different emergency care pathways. Wider factors influencing emergency hospital use in cancer patients lay in what is personal, what goes on with care close to home, what happens in the hospital, and the junctures of these. Our study is the first to have identified and mapped interactions and feedback loops among comprehensive factors relating to main players and main parts of the care system, drawing on a Systems thinking tool- CLDs. This facilitated a whole-system, integrated understanding of the drivers of and solutions to the increasing pressure on emergency hospital care- highly relevant to service planners.

Our study adds to the limited evidence on the benefits of specialised, urgent and emergency cancer care. Particularly, we have identified a comprehensive emergency cancer care model which optimised care of acutely ill cancer patients on different care pathways, i.e. those presenting to the cancer centre and those to the acute general hospital *via* A&E. This model of care functioned to a) prevent unnecessary hospital use by supporting patients, carers and community-based professionals to (self) manage acute symptoms and use appropriate services in the community (the help line); b) if hospital use is necessary, triage patients to the most appropriate (ambulatory or inpatient) service (the help line); and c) improve patient outcome and experience in the hospital by providing prompt and effective care directly (the walk-in clinic and the wards) or by supporting non-cancer specialists in clinical decision making about acute complex problems in the acute hospital (the in-reach service). The core element of this model is the prompt access to senior cancer specialists by patients, carers and other health professionals who care for cancer patients. Our findings support those about general emergency care that there should be senior responsibility for the patient and the clinical management plan from emergency admission to discharge because it improves patient outcomes, reduces admissions rates, length of stay and costs of care; and that ambulatory emergency care is clinically safe and reduces pressure on beds [3,29]. However, our findings highlight the need for not just senior but also *clinically appropriate* specialist responsibility for the patient and the clinical decision-making in emergency care.

Despite its effectiveness in preventing avoidable emergency hospital use and managing the unavoidable, the cancer centre’s specialised emergency cancer care is only one piece of the jigsaw. We found other important factors simultaneously influencing emergency hospital use- related to patients and carers, primary, secondary and community health care, social care and interfaces. Our findings add to the growing evidence supporting that a) providing timely access to GP [30,31], b) improving lay self-care and service navigation/utilisation abilities [32–35], and c) meeting individuals’ social care needs [2,35–37] help to reduce emergency hospital use for people with any condition. Moreover, we identified the need for community health professionals to be supported by cancer specialists with regard to knowledge, skills and confidence in managing cancer patients particularly when they were acutely ill. For cancer patients approaching end of life, our findings support the existing evidence that early EOL discussions e.g. ACP and DNA-CPR [38,39], and availability, accessibility and quality of care close to home (GP, district nursing, social care, specialised palliative care) and earlier involvement and collaboration between oncology and palliative care [40–43], reduce the odds of cancer patients receiving inappropriate aggressive EOL care: chemotherapy, ED visits, ICU care, emergency admissions, long stay and hospital death.

In secondary care, we found that it was critical that cancer patients got to the right place (assessment unit, ambulatory care or ward) for the right care (right facilities, processes and expertise) at the right time (no delays and extra referrals and transfers). This ensured that patients had their acute care and other special needs met in a timely manner. Bed capacity was identified as a major hindrance to patients being admitted to the right ward. In this trust and indeed in almost all UK hospitals, patients are regularly placed on wards that are clinically suboptimal if there are no beds available on the right specialty ward [44]. This practice was found to a) create competing demands on staff members’ time resulting in delays, b) pose communication barriers compromising input from knowledgeable staff, c) provide an unsuitable ward environment and, and d) be inappropriate for patients’ needs. As the policy trend is to decrease the overall number of beds, alternative measures are necessary to resolve this problem, such as the acute oncology in-reach service in this study, which mitigated the above issues to some extent. Our data also support Friebel and Steventon’s [45] finding that some re-admissions are preventable by making improvements to the quality and safety of the initial hospital stay, transitional care, and post-discharge support. However, coordination of and handovers among complex care services were identified as the most challenging part of discharge particularly due to social care crisis.

Within and across all settings, timely and effective sharing of key patient information and effective communication among professionals and between them and lay people also played a role in emergency hospital use in cancer patients. This is because these underpin care coordination and continuity of care- fundamental to safe, effective, efficient patient care [3,46]. Royal College of Physicians and Royal College of Radiologists recommend that information about a patient, revised at all key points in the cancer journey, should be available 24 hours a day, 7 days a week to all healthcare professionals who may encounter that patient if they present with acute care needs in any care setting [6]. An additional new finding of our study is that professionals also lacked knowledge about or skills in accessing the available services in the same and different settings. This may hinder patients getting the right care at the right place and right time, leading to avoidable hospital use or worse outcome and experience in the hospital.

### Clinical implications

We pulled together all the influencing factors that we found using CLDs (Figs 3 and 4), to achieve a whole-system, integrated understanding of the complexity and dynamics in emergency cancer care. We identified five reinforcing feedback loops revolving around eight factors (see Fig 4). We focused on virtuous cycles represented by these loops. Together, they show that a) reduction of avoidable hospital use is of crucial importance because it helps relieve hospital bed pressure; b) improved bed capacity then has a decisive, positive influence on patient pathway and thus experience and outcome in the hospital; c) in turn, better in-hospital care and discharge help patients and carers self-care and cope better back home with better support from community-based health and social care services, which then reduces their future emergency hospital use. The eight factors forming these loops are therefore of high-leverage influence as they add to each other along the loops, and through which the self-propagating ability of the loops can be further strengthened or weakened by factors directly or indirectly connected to them. This helps to explain why “timely access to (senior) cancer specialists for advice, assessment, clinical decision & support”–hence the cancer centre’s comprehensive emergency cancer care services, is essential in emergency cancer care: it can augment the virtuous circles by improving: lay self-care and service utilisation abilities, management of ACSCs by primary and community care, patient pathway to right ambulatory or inpatient care, prompt and effective management and prevention of acute problems, and discharge success. Our CLDs also highlight the need for all parties and players of the cancer care system to make their contribution in a mutually supportive way- to maintain or strengthen the virtuous circles- eventually to benefit themselves. In terms of vicious circles represented by our CLDs, a good example is that funding cut in one part as often mentioned by our participants (e.g. hospital bed, GP services or social care) impairs not only capacity and quality of care in the targeted part but also other parts of the care system and even create a downward spiral affecting the whole system. Our CLDs thus can facilitate identification and understanding of unintended consequences and unexpected phenomena of interventions, practices and policies and can be useful for service planners to guide focus to key influencers.

## Limitations

Our CLDs are context-dependent, yet they do not capture the context. Therefore they must be understood with the accompanying narratives. They represent the mental model of the secondary care professionals. Other stakeholders of the emergency cancer care system, e.g. community-based professionals and patients and carers, were not included in the study. Their experiences and views may differ. For example, in our previous study [4], although patients and carers reported not getting sufficient primary and community health care, they did not attribute their emergency admissions to this. We found in this study that health professionals may not know all the services in the same setting, not to mention in other settings. For example, GPs may not have the same understanding of the hospital services; however they may know more about what happens in the community: community-related factors influencing emergency admissions and community-based practices and interventions to reduce admissions. Including the views of these stakeholders may change the result, which warrants future research to incorporate their views into the model. Similarly, the non-respondents may also have different experiences and views. However, our sample size (42) is quite large for a qualitative study and we interviewed as many professionals as data saturation required. Moreover, the respondents were core professionals in charge of the care of acutely ill cancer patients on the main emergency care pathways; as key clinical decision maker, supervisor, advisor and/or leader, they were able to provide comprehensive and sophisticated expert knowledge regarding the matters under investigation. As such, it is safe to assume that the missing views of the non-respondents may not yield significant changes to the CLDs.

The data were not intended for CLDs development originally. Although participants discussed the underlying causes of the problem under investigation, it was likely that such discussions were not exhausted with some participants. Therefore, there may be missing causal factors or missing links. There may also be non-causal relationships included- researchers might have erroneously attributed causality. While it is not ideal to have missing factors, including everything in the diagrams compromises their power to communicate complexity. So a balance was needed between comprehensiveness and clarity. To reduce cluttering and enhance clarity, we had to aggregate and abstract variables and prune non-essential links, risking losing nuances or leaving out factors and links. We made up for this as best as possible by providing detailed and nuanced descriptions and explanations about variables. The modeller HC did not collect data, nor were the CLDs verified with the original stakeholders due to funding and time constraints. The distance between data source and modeller mean that biases may be introduced into the CLDs. The systematic coding and documenting method [28] allowed the modeller to leave a trace of data–CLD linkage and, where feasible, created an opportunity for the CLDs to be examined by members of the research team. Thus it helped to reduce biases.

## Conclusions

For acutely ill cancer patients, it is essential that patients, carers and other health professionals sharing responsibility for cancer patients have prompt access to senior cancer specialists for advice, assessment, clinical decision and other support. It helps reduce avoidable presentations and admissions and length of hospital stay while improving patient outcome and experience on different emergency care pathways. However, drivers of emergency hospital use in cancer patients are complex relating to different parts and players of the cancer care system. Our CLDs captured interactions and feedback loops among these drivers and facilitated a whole-system, integrated understanding of the complexity and dynamics in emergency cancer care. They can be used to inform policy and intervention design and service planning and evaluation beyond immediate effects but extend to unintended and unexpected ripple effects.
